# Bioinformatic Analysis of Genetic Factors from Human Blood Samples and Postmortem Brains in Parkinson's Disease

**DOI:** 10.1155/2022/9235358

**Published:** 2022-12-24

**Authors:** Longping Yao, Kai Lin, Zijian Zheng, Sumeyye Koc, Shizhong Zhang, Guohui Lu, Thomas Skutella

**Affiliations:** ^1^Department of Neuroanatomy, Group for Regeneration and Reprogramming, Institute for Anatomy and Cell Biology, Medical Faculty, Heidelberg University, 69120 Heidelberg, Germany; ^2^Nursing Department of Zengcheng Branch, Nanfang Hospital, No. 28 Innovation Avenue, Zengcheng, Guangzhou 511300, China; ^3^Department of Neurosurgery, First Affiliated Hospital of Nanchang University, 330006 Nanchang, China; ^4^Department of Neuroscience, Institute of Health Sciences, Ondokuz Mayıs University, 55200 Samsun, Turkey; ^5^Department of Neurosurgery, Southern Medical University, 510280 Guangzhou, China

## Abstract

Parkinson's disease (PD) is one of the most prevalent neurodegenerative disorders characterized by motor and nonmotor symptoms due to the selective loss of midbrain dopaminergic neurons. Pharmacological and surgical interventions have not been possible to cure PD; however, the cause of neurodegeneration remains unclear. Here, we performed and tested a multitiered bioinformatic analysis using the GEO and Proteinexchange database to investigate the gene expression involved in the pathogenesis of PD. Then we further validated individual differences in gene expression in whole blood samples that we collected in the clinic. We also made an interaction analysis and prediction for these genetic factors. There were in all 1045 genes expressing differently in PD compared with the healthy control group. Protein-protein interaction (PPI) networks showed 10 top hub genes: ACO2, MDH2, SDHA, ATP5A1, UQCRC2, PDHB, SUCLG1, NDUFS3, UQCRC1, and ATP5C1. We validated the ten hub gene expression in clinical PD patients and showed the expression of MDH2 was significantly different compared with healthy control. Besides, we also identified the expression of G6PD, GRID2, RIPK2, CUL4B, BCL6, MRPS31, GPI, and MAP 2 K1 were all significantly increased, and levels of MAPK, ELAVL1, RAB14, KLF9, ARF1, ARFGAP1, ATG7, ABCA7, SFT2D2, E2F2, MAPK7, and UHRF1 were all significantly decreased in PD. Among them, to our knowledge, we presently have the most recent and conclusive evidence that GRID2, RIPK2, CUL4B, E2F2, and ABCA7 are possible PD indicators. We confirmed several genetic factors which may be involved in the pathogenesis of PD. They could be promising markers for discriminating the PD and potential factors that may affect PD development.

## 1. Introduction

Parkinson's disease (PD) is an age-related progressive neurodegenerative disorder caused by selective loss of midbrain dopaminergic (DA) neurons in the substantia nigra pars compacta (SNpc) [1–5]. It has been a worldwide public health problem characterized by motor and nonmotor symptoms [6, 7]. Lewy bodies, protein aggregates containing *α*-synuclein, are often deposited in several brain areas of people with PD [8]. Motor symptoms, primarily dependent on dopaminergic nigrostriatal denervation, gradually manifest as DA neuron survival decreases [1]. People with PD also have sleep issues, exhaustion, changed mood, cognitive problems, autonomic dysfunction, and pain as the illness progresses and neurodegeneration worsens [9]. These symptoms result from alterations occurring at various levels of the brain. The primary pathogenic alteration is the gradual degradation of neurons in the substantia nigra pars compacta, one of the basal ganglia's nuclei [10]. These neurons are involved in the transmission of dopamine to the striatum and another basal ganglia nucleus. The neural circuits, which comprise the basal ganglia and motor cortical regions, become dysfunctional due to the degeneration of these neurons [10]. In the end, these modifications to behavior at the level of an individual lead to movement irregularities, the main symptoms of PD, which impact the quality of life of the person. Currently, pharmacological and surgical treatment cannot to prevent or cure this disease [11]; however, understanding of neurodegeneration remains poor.

To date, some genetic biomarkers have been reported to be associated with the pathogenesis of PD [12]; such as *α*-synuclein, glucocerebrosidase, leucine-rich repeat kinase 2, and synaptojanin 1, can be useful to diagnose or assess the risk of PD [10]. However, these factors can only partly explain the pathogenesis of PD in some patients. Thus, further studies on the genetic architecture of PD are urgently needed. Transcriptomics and proteomics studies have been widely used to explore neurogenesis in PD [13, 14]. However, single-chip testing cannot fully reflect the complete genetic spectrum of PD because of the small sample size and differences in sample populations. In this study, we aimed to discover some potential markers for discriminating the PD and potential factors affecting PD development. Here, we combined the datasets from Gene Expression Omnibus (GEO) and Proteinexchange and validated the genes associated with PD. We analyzed the variable genes with weighted gene coexpression network analysis (WGCNA), Kyoto Encyclopedia of Genes and Genomes (KEGG) functional enrichment analyses (GOFEA, KEGGFEA), Gene Ontology (GO), and STRING protein-interacting network and confirmed hub interacting proteins/genes expression, which showed the metabolic pathways were tightly related in the pathogenic and pathogenesis. Further, we also verified that the expression of GRID2, PADI4, RIPK2, CUL4B, BCL6, MRPS31, GPI, and MAP 2 K1 were both significantly increased, and levels of MAPK, ELAVL1, RAB14, KLF9, ARF1, ARFGAP1, ATG7, ABCA7, SFT2D2, E2F2, MAPK7, and UHRF1 were both significantly decreased in PD.

## 2. Materials and Methods

### 2.1. Data Acquisition and Processing

**Figure 1:**
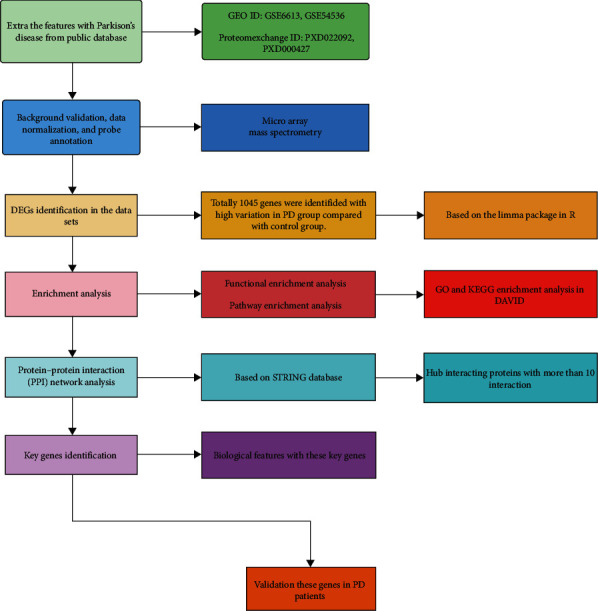
Study flow diagram.

Datasets were employed from Gene Expression Omnibus (GEO) public database (https://www.ncbi.nlm.nih.gov/gds) and Proteomexchange public database (http://www.proteomexchange.org/) for the keywords “Parkinson's disease” and “PD.” The R software has been used for sample expression matrix acquisition background correction, data downgrade extraction, normalization, log_2_ transformation, and probe reannotation. R packages that were applied in this study were *RMA, devtools, AnnoProbe, limma, Biobase, affyPLM,* and *GEOquery*. If a single gene in the chip corresponded to numerous probes, the expression level of the gene was computed using the average value of these multiple probes. *Using* Fisher's combined probability test, *P* values were pooled, then adjusted for multiple comparison correction using the false discovery rate (FDR). The significance level was established at FDR ≤ 0.05. The study workflow diagram of analysis for transcriptomic and proteomics was concluded, and the bioinformatics analysis for differential expressed genes and case–control study flow diagram was shown in Figure 1.

Mass spectrometry is a very central analytical technique for protein research and the study of biomolecules in general [15, 16]. It can be used to identify, characterize, and quantify proteins at ever-increasing sensitivity and ever more complex samples [15]. The sequence of amino acids of proteins provides contact between proteins and their coding genes via the genetic code, and, in principle, a link between cell physiology and genetics. The discovery of proteins opens a glimpse into intricate biological regulatory networks [15]. The mass spectrometry data was analyzed by Xcalibur 2.2 data processing software (Thermo Fisher Scientific Inc.). The raw data require conversion from raw format (.raw) into text format (.txt). This phase could take a few minutes depending on the quantity of files that need to be converted and the computer's capacity to handle big volumes of data.

### 2.2. WGCNA and Hub Genes Selection

WGCNA is a commonly used data mining method that uses pairwise correlations between variables to analyze biological networks [17]. While it can be used to analyze various high-dimensional data sets, it is most commonly utilized in genomics. It allows researchers to define modules (clusters), intramodular hubs, and network nodes based on module membership, examine coexpression module interactions and compare network topologies (differential network analysis). Correlation networks make it easier to apply network-based gene screening approaches to find potential biomarkers or therapeutic targets. The weighted correlation network analysis was carried out using the R package, WGCNA. We estimated the dissimilarity of the modules, determined a cut line for the module dendrogram, and merged some modules to examine the modules further. We also merged the modules with a distance of less than 0.35, yielding 5 coexpression modules in the end.

### 2.3. Cluster Analysis

ConsensusClusterPlus [18] was used to perform cluster analysis, including agglomerative pam clustering with 1-Pearson correlation distances and resampling 80 percent of the samples for 10 repetitions. The empirical cumulative distribution function plot was used to find the appropriate number of clusters.

### 2.4. Functional Enrichment Analysis

We used DAVID (https://david.ncifcrf.gov/home.jsp) to run GO and KEGG functional enrichment studies to find the most highly enriched pathways, revealing the module's potential biological importance. The *P* values were calculated using Fisher's exact probability approach to obtain statistically significant gene sets with meaningful functional annotations and signaling pathways. The significance level was chosen at P < 0.05.

### 2.5. Protein-Protein Interaction Network and Hub Genes Selection in the Key Module

The STRING 11.5 database (https://string-db.org/cgi/input?sessionId=boTaerIW4Ew9&input_page_show_search=on) was used to map the protein-protein interaction (PPI) network and predict the interactions of all of the proteins discovered in the study. The activity of PPI is a primary focus of cellular biology research and serves as a prerequisite for system biology [19]. Proteins interact with other proteins inside the cell to fulfill their functions, and information generated by a PPI network enhances the perception of the protein's function [20]. To create a network map of PPI, we used the STRING database's data analysis mode and an integrated confidence value of 0.4. The STRING platform was used to calculate the confidence score, which is a medium confidence score. The acquired PPI are evaluated using the software Cytoscape (https://cytoscape.org/) for a better visual depiction of the network and to identify hub genes [21].

### 2.6. Reverse Transcription-Quantitative Real-Time Polymerase Chain Reaction

Clinical whole blood samples with PD patients and healthy control were collected. Total RNA was extracted according to the manufacturer's instructions using TRIzol reagent (15596018; Invitrogen, Carlsbad, CA, USA). RNA was reverse transcribed to complementary DNA (cDNA) with a random primer (Sangon Biotech, Shanghai, China) using a Reverse Transcription Kit (RR047A; Takara, Dalian, China) for the quantification of messenger RNA (mRNA) of protein-encoding genes and the mRNA levels were determined using reverse transcription-quantitative real-time polymerase chain reaction (RT-qPCR). The internal control was a human glyceraldehyde-3-phosphate dehydrogenase (GAPDH) primer pair. The Ct technique ∆∆*C_t_* method was used to calculate and normalize relative gene expressions [22, 23]. All the sequences of the primers used are listed in Table 1.

### 2.7. Case Study Design

The ethics committee of Zhujiang Hospital of Southern Medical University approved this study. After receiving informed consent, a total of 40 PD patients from Zhujiang Hospital of Southern Medical University and 21 healthy controls who had not been diagnosed with a central nervous system disease in the past. The following topics were not included: (1) patients with Parkinson's disease brought on by cardiovascular diseases, cerebrovascular illness, encephalopathy, trauma, tumors, or medications; (2) patients with various degenerative diseases, such as corticobasal degeneration, Huntington's disease, dementia with Lewy bodies, and Alzheimer disease (AD); (3) people with other central nervous system illnesses, such as cerebrovascular disease, intracranial infection, other central nervous systemic autoimmune diseases, demyelinating disease, trauma, malignancy, toxic diseases, and metabolic diseases; (4) patients with somatic impairments, such as aphasia, severe dementia or consciousness disturbance, cancer, renal failure, hepatic failure, cardiopathy, and any other acute or chronic incapacitating or life-threatening disease/state; and (5) patients who declined to participate in the research.

The 40 peripheral blood samples of PD patients (male: 25 cases and female: 15 cases) and 21 healthy controls (male: 14 cases and female: 7 cases) were collected by the experienced nurse in the hospital. The information on sex, age, onset time, and complications was recorded. Within two hours after blood collection, blood samples were produced by centrifuging at 1200 g for 10 min at 20°C in a mini-41 C centrifuge (Heima Medical Apparatus Company, Zhuhai, China). RNA total was isolated. Based on our bioinformatic analyses, the genes whose FDR ≤ 0.001 and hub genes were selected for QT-qPCR validation.

### 2.8. Statistical Analysis

The results were provided as the mean SD of three separate studies. A two-tailed Student's *t*-test was used in the statistical analysis. A statistically significant difference was defined as one with *P* < 0.05.

## 3. Results

### 3.1. Consensus Clustering Provides Quantitative Evidence for Determining the Number and Membership of Possible Clusters in the Datasets

**Figure 2:**
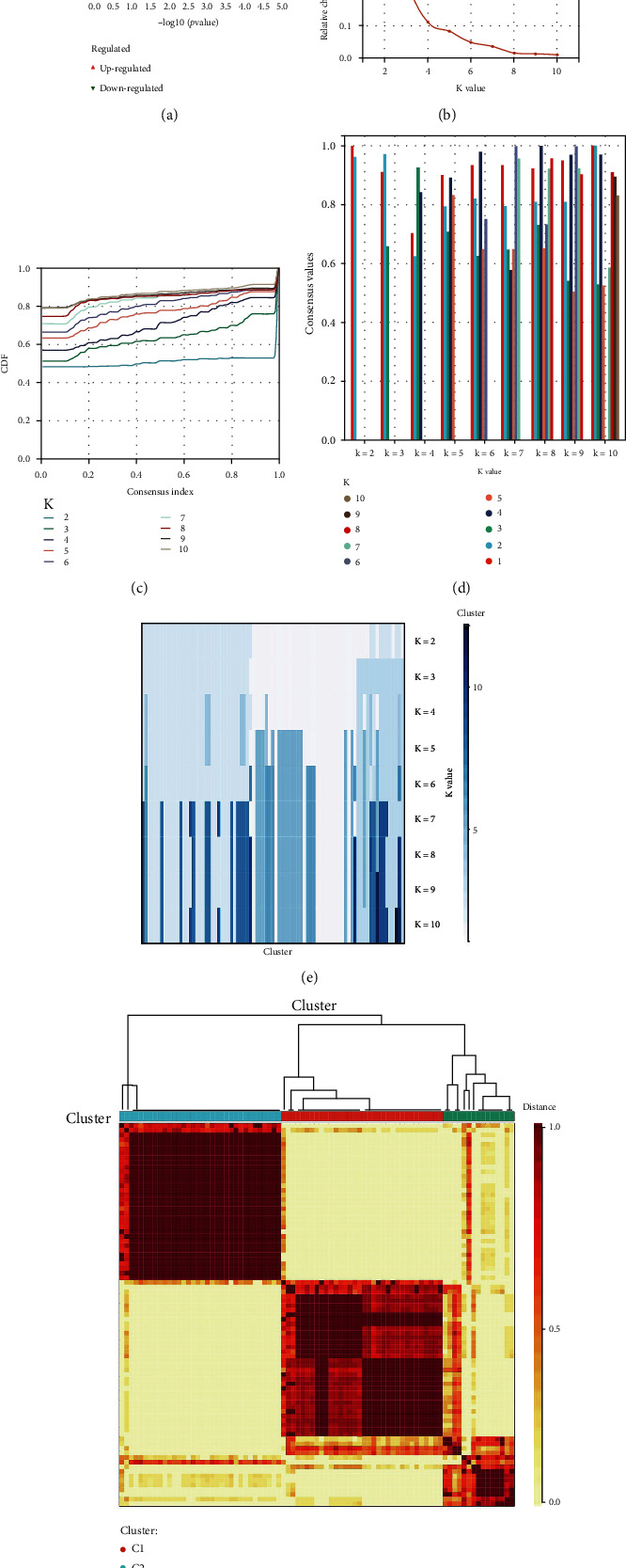
Consensus clustering provides quantitative evidence for determining the number and membership of possible clusters in the datasets. (a) Volcano plots. (b) CDF curve. (c, d) Consensus clustering delta area curve (c) and column chart (d) displaying the relative change in area under the cumulative distribution function (CDF) curve for each category number *k* compared to *k*–1. The vertical axis depicts the relative change in area under the CDF curve, while the horizontal axis reflects the category number k. (e, f, d) Consensus *k* = 2 sample cluster heat map. Different samples are represented by the rows and columns of the matrix. The values of the consensus matrix range from 0 (cannot be clustered together) to 1 (always clustered together) in yellow to dark red.

The GEO database has 14 PD-related microarray transcriptome datasets, and the Proteinexchange database has 45 PD-related mass spectrometry (MS) proteomics datasets. This study processed the datasets with human whole blood samples and post-mortem substantia nigra pars compacta (SNpc). Besides, each dataset group should have at least 3 or more duplicates; what is more, a healthy control group should be included in the inclusive dataset. Based on the above inclusion criteria, 2 GEO datasets and 2 Proteinexchange datasets were finally applied in this study (Table 2). 1045 differentially expressed genes (DEGs) were identified with the threshold of FDR ≤ 0.05. These DEGs' differential expression profiling and distribution were displayed (Figure 2(a)). We used the ConsensusClusterPlus package to perform cluster analysis, which included agglomerative pam clustering with 1-Pearson correlation distances and resampling 80 percent of the samples for 10 repetitions. The Cumulative Distribution Function (CDF) identified the best cluster number. The cluster was found to be the best stable clustering based on the CDF Delta area curve when *k* = 2 (Figures 2(b), 2(c), and 2(d)). We used *k* = 2 to get four Immune Subtype (IS) results (Figures 2(e) and 2(f)).

### 3.2. WGCNA Analysis the Genes which Expressed Differentially

**Figure 3:**
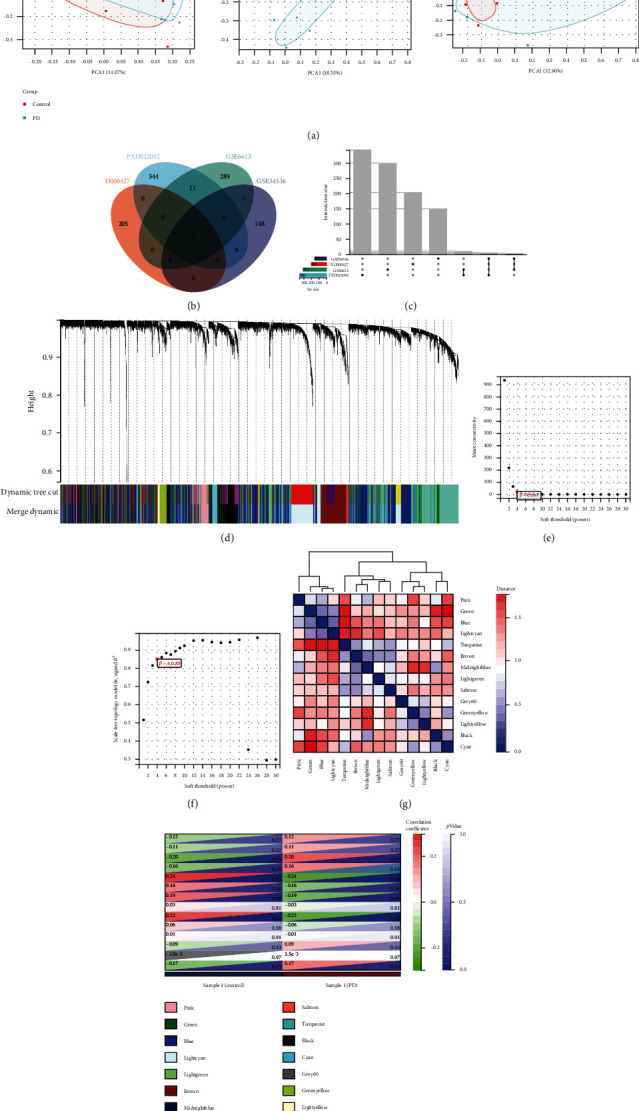
WGCNA analysis of the genes that expressed differentially. (a) The MetaQC software package of R was used to do the PCA analysis for each dataset. (b, c) The Venn diagrams of the four datasets. (d) 1-TOM cluster has been used to create a tree of all gene expressions. (e) Soft Threshold (Power) reflects the weight, while the vertical axis depicts the network's average connectedness. (f) The weight is represented by Soft Threshold (Power), and the scale-free topology fitting index *R*^2 is shown on the vertical axis. (g) The heat maps of correlations between modules show genes and samples, with correlation coefficients and *P* values in each cell. (h). The heat maps of correlations between modules show genes and samples, grouped into PD and control, with correlation coefficients and *P* values in each cell.

Each of the datasets was identified by principal component analysis (PCA) (Figure 3(a)). Different databases contribute differently to the total number of proteins, with varying degrees of database overlap (Figures 3(b) and 3(c)). The 1045 DEGs were hierarchically classified into 14 categories using WGCNA analysis, respectively, black, blue, brown, cyan, green, greenyellow, grey60, lightcyan, lightgreen, lightyellow, pink, royalblue, salmon, and turquoise (Figure 3(d)). We used the network's average connectivity (Figure 3(e)) as the horizontal axis and the scale-free topology fitting index *R*^2 (values in the SFT.R.Sq column in the statistical data) as the vertical axis (Figure 3(f)). In the heat map of correlation between the tree diagram of gene expression and module features, we discovered 14 modules as a result of resprung of the cluster tree (Figure 3(g)). The grouping of PD and control displayed the relationship of modules and a phenotype heat map, and the greenyellow module had the most robust inverse relationship with the PD phenotype (Figure 3(h)).

### 3.3. GO and KEGG to Identify the most Highly Enriched Pathways

**Figure 4:**
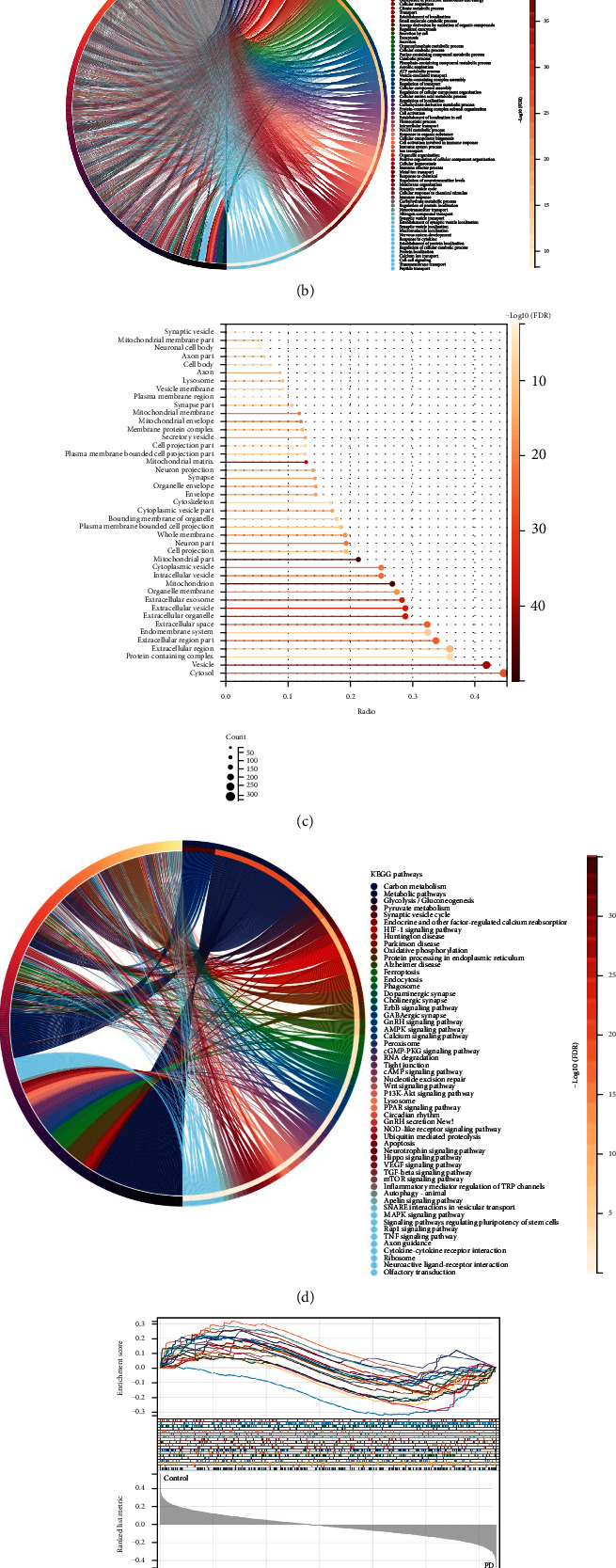
GO and KEGG to identify the most highly enriched pathways. (a) Network of enriched terms: (a) colored by cluster-ID, where nodes that share the same cluster-ID are typically close to each other; (b) colored by *P* value, where terms containing more genes tend to have a more significant *P* value. (b) Gene list enrichments are identified in the biological progress (BP) category, and (c) in the cellular component (CC) category. As an enrichment background, all genes in the genome were employed. Terms with a *P* value of less than 0.01, a minimum count of three, and an enrichment factor of more than 1.5 (the enrichment factor is the ratio between the observed counts and the counts expected by chance) are gathered and classified into clusters based on membership commonalities. (d, e) KEGG biological pathways analysis of 1045 which is involved in PD.

The DAVID was used to conduct GO and KEGG analyses to further investigate the biological processes and pathways involved (Table 3) [24]. According to GO enrichment analysis, the 1045 genes were primarily enriched in the biological progress (BP) category for the oxidation-reduction process, molecule, and metabolic drug process, cellular respiration, immune system process, regulated exocytosis, transport, the establishment of localization, and cellular respiration; the cellular component (CC) category for cytosol, vesicle, protein-containing complex, extracellular region, organelle membrane, extracellular exosome, extracellular vesicle, extracellular organelle, and endomembrane system (Figures 4(a), 4(b), and 4(c)). Many transcription factors were enriched, which were associated with PD (Table 4). The 1045 genes were mostly enriched in carbon metabolism, metabolic pathways, glycolysis/gluconeogenesis, pyruvate metabolism, synaptic vesicle cycle, endocrine, and other factor-regulated calcium reabsorption, HIF-1 signaling pathway, Huntington's disease, PD, oxidative phosphorylation, protein processing in the endoplasmic reticulum, and Alzheimer's disease (AD) according to KEGG pathway analysis (Figures 4(d) and 4(e)).

### 3.4. The PPI Network Reveals Interacting Proteins and Hub Genes

**Figure 5:**
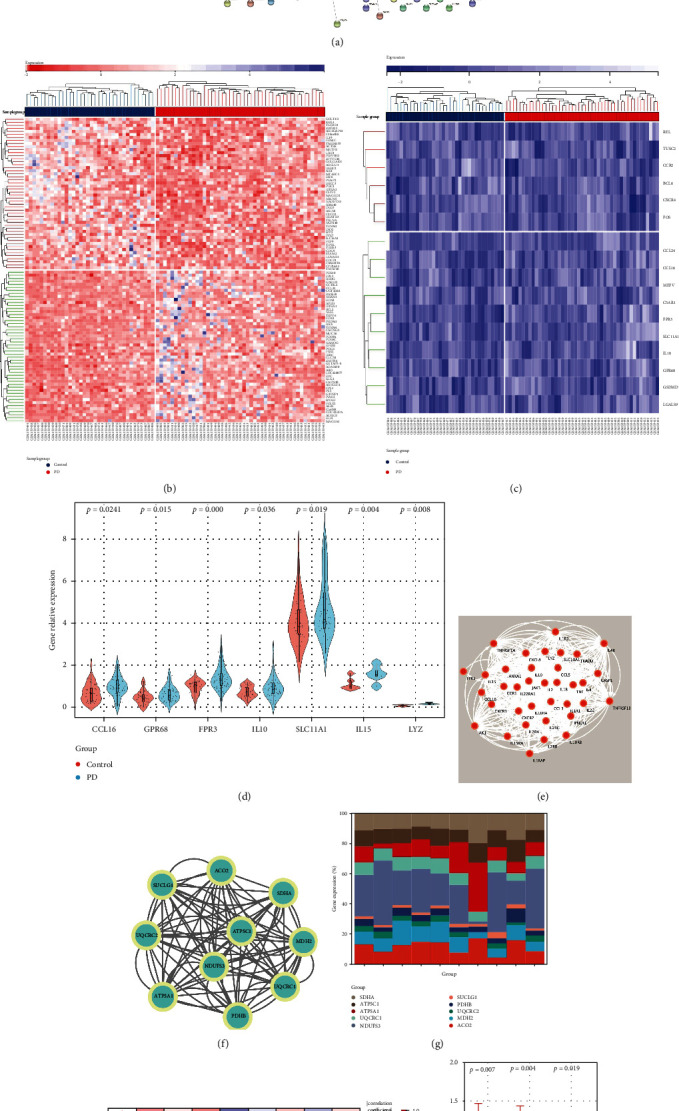
The PPI network reveals interacting proteins. (a) The STRING web program created a PPI network for the genes in DEGs. There were 600 protein-protein interactions in total. (b) Heat map displayed the 50 genes that showed clear alterations in PD. (c) Heat map shows the expression of inflammation-related genes in PD and control groups. (d) Relative gene expression of CCL16, GPR68, FPR3, IL10, SLC11A1, IL15, and LYZ increased significantly in PD compared with the control group. Data are shown as mean ± se. (e) The STRING web program created a PPI network for the inflammation-related genes. (f) The STRING web program created a PPI network for the hub genes to show the interaction between the proteins. (g) The relative hub genes levels of ACO2, MDH2, SDHA, ATP5A1, UQCRC2, PDHB, SUCLG1, NDUFS3, UQCRC1, and ATP5C1 in PD and control group. (h) Pearson correlation coefficients among individual 10 hub genes. (i) The relative expression of top 3 hub genes ACO2, MDH2, and UQCRC1 in PD and control group.

STRING 11.5 was used to input the frequent DEGs, and the file generated from the analysis was reintroduced into Cytoscape for further investigation of this study, including hub gene detection [25, 26]. The PPI network investigated gene-gene/protein-protein interactions, identifying 600 pairs of interactions with a medium confidence interaction score (0.4) (Figure 5(a)). In the heat map, we chose 50 genes that showed evident alterations in PD compared with the control (Figure 5(b)). Besides, our previous research proposes a mechanism underlying neurodegeneration in chronic CNS inflammation caused by microglial activation [22, 23]. This study further confirmed that many inflammation-related genes, such as CCL16, GPR68, FPR3, IL10, SLC11A1, IL15, and LYZ, have undergone extensive changes (Figures 5(c) and 5(d)). The PPI network was utilized with a medium confidence interaction score to investigate gene-gene/protein-protein interactions with inflammation-related genes (0.4) (Figure 5(e)). Then, hub genes and essential modules were detected based on the PPIs network in Figure 5(a). We selected the top 10 hub genes of ACO2, MDH2, SDHA, ATP5A1, UQCRC2, PDHB, SUCLG1, NDUFS3, UQCRC1, and ATP5C1 and analyzed their interactions (Figure 5(f)), and the relative expressions of the interacting proteins were shown in Figure 5(g). The expressions between NDUFS3 and UQCRC2, UQCRC1, and UQCRC2 had a significant positive correlation; meanwhile, ATP5C1 and ATP5A1 expressed a high negative correlation (Figure 5(h)). The top four hub node interacting genes' relative expressions were ACO2, MDH2, and UQCRC1 (Figure 5(i)).

### 3.5. A Case-Control Study Was Used to Validate Peripheral Blood PD Markers

**Figure 6:**
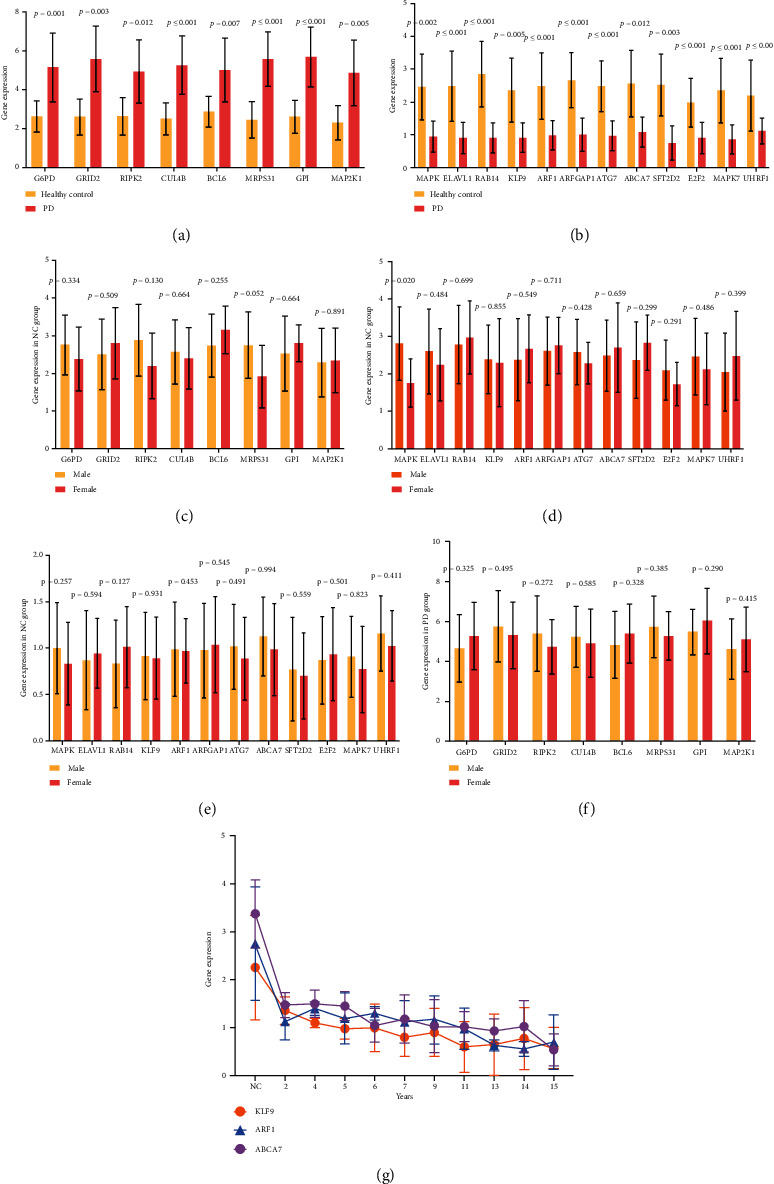
A case-control study was used to validate peripheral blood PD markers. (a) The mRNA expression levels of G6PD, MDH2, GRID2, RIPK2, CUL4B, BCL6, MRPS31, GPI, and MAP 2 K1 were studied using qRT-PCR in a total of 40 PD patients and 21 healthy controls. (b) The mRNA expression levels of MAPK, ELAVL1, RAB14, KLF9, ARF1, ARFGAP1, ATG7, ABCA7, SFT2D2, E2F2, MAPK7, and UHRF1 were evaluated by qRT-PCR. (c–f) The mRNA expression of G6PD, MDH2, GRID2, RIPK2, CUL4B, BCL6, MRPS31, GPI, MAP 2 K1, MAPK, ELAVL1, RAB14, KLF9, ARF1, ARFGAP1, ATG7, ABCA7, SFT2D2, E2F2, MAPK7, and UHRF1 were compared between healthy control and PD patients. (g) The mRNA levels of KLF9, ARF1, and ABCA7 were shown in a time-dependent matter.

RT-qPCR analyzed the peripheral blood samples of PD and healthy control to measure the gene expression. We selected the genes whose FDR ≤ 0.001 and hub genes for further validation. Totally 132 genes matched the criterion. We measured 132 genes expression in blood samples of PD patients. As a result, the levels of G6PD, GRID2, RIPK2, CUL4B, BCL6, MRPS31, GPI, and MAP 2 K1 were much higher in PD than in the healthy control (Figure 6(a)). What is more, the genes expression of MAPK, ELAVL1, RAB14, KLF9, ARF1, ARFGAP1, ATG7, ABCA7, SFT2D2, E2F2, MAPK7, and UHRF1 were both significantly decreased in PD (Figure 6(b)). Meanwhile, there was no difference in these genes' expression between the male and female group in both healthy control and PD patients, except for MAPK in healthy control (*P* = 0.020) (Figures 6(c), 6(d), 6(e), and 6(f)). Moreover, we found that KLF9, ARF1, and ABCA7 showed a decreasing trend with the increase in disease duration (Figure 6(g)).

### 3.6. The PPI Network Reveals Interaction for the Peripheral Blood PD Markers

**Figure 7:**
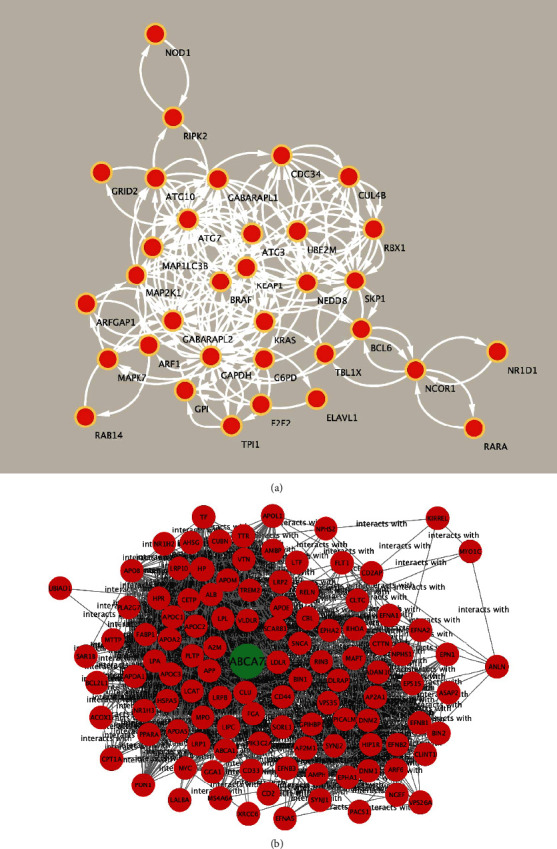
The PPI network reveals interaction for the peripheral blood PD markers. (a) The STRING web program created a PPI network for the clinically validated peripheral blood PD markers. (b) The STRING web program created a PPI network for the gene of ABCA7.

A PPI network for the genes of G6PD, MDH2, GRID2, RIPK2, CUL4B, BCL6, MRPS31, GPI, MAP 2 K1, MAPK, ELAVL1, RAB14, KLF9, ARF1, ARFGAP1, ATG7, ABCA7, SFT2D2, E2F2, MAPK7, and UHRF1 was built using the STRING online tool (Figure 7(a)). The PPIs network contains a number of nodes: 40, number of edges: 91, and average node degree: 4.55. Among these factors, the ATP-binding cassette, subfamily A, member 7 (ABCA7) gene has one of the highest post-GWAS research success rates, with the discovery of both common risk variants with a direct functional consequence on ABCA7 and rare coding variants of intermediate to high penetrance providing compelling evidence of ABCA7's involvement in AD risk [27, 28]. In this investigation, we discovered that the expression of ABCA7 in PD was dramatically reduced, implying that abnormal ABCA7 expression is linked to the pathogenic process of PD. Currently, no one has yet published this finding in PD. The STRING online tool was used to create a PPI network for ABCA7, number of nodes: 40, number of edges: 91, and average node degree: 4.55. (Figure 7(b)).

## 4. Discussion

PD is the second most common neurodegenerative ailment after AD, and it has become a major public health concern globally [1, 29]. Despite new proposals for numerous pathogenetic processes, the cause of neurodegeneration remains unexplained, and preventing or curing the disease is not possible at present [30]. Integrating data from various sources has previously been used to find genes with consistently high significance scores across multiple studies [31, 32].

In this study, we performed and tested a multitiered bioinformatic analysis using the GEO and Proteinexchange database to investigate the gene expression involved in the pathogenesis of PD. Because of their ease of availability compared to those from cerebrospinal fluid (SCF), molecular profiles are increasingly being employed as diagnostic biomarkers for PD as genomics and transcriptomics progress in human complex disease. An increasing variety of annotation resources is now available to prioritize and filter genomic and proteome variants. Here, we introduced the recently developed WGCNA methodology, a commonly used data mining method based on pairwise correlations between variables and is especially useful for investigating biological networks [33]. These genetic factors were also subjected to an interaction analysis and prediction.

Totally, 1045 genes were expressed differently in people with PD compared to those in the healthy control group. The following ten top hub genes were discovered in protein-protein interaction (PPI) networks: ACO2, MDH2, SDHA, ATP5A1, UQCRC2, PDHB, SUCLG1, NDUFS3, UQCRC1, and ATP5C1. Then we further validated differences in gene expression in whole blood samples collected in the clinic. MDH2, one of the key hub genes, established that the expression had significantly risen in PD compared with the control. Besides, we also identified the levels of genes G6PD, GRID2, RIPK2, CUL4B, BCL6, MRPS31, GPI, and MAP 2 K1 were both significantly increased, and levels of genes MAPK, ELAVL1, RAB14, KLF9, ARF1, ARFGAP1, ATG7, ABCA7, SFT2D2, E2F2, MAPK7, and UHRF1 were both significantly decreased in PD.

Our findings imply that these indicators, verified using clinical whole blood samples, could be used as a biomarker to distinguish between PD cases and controls. Among them, to our knowledge, we presently have the most recent and conclusive evidence that GRID2, RIPK2, CUL4B, E2F2, and ABCA7 are possible PD indicators. ABCA7 was newly identified in the early 2000s by Kaminski et al. and colleagues from human macrophages [34]. The expression of ABCA7 in PD is dramatically reduced according to our findings from the validation of peripheral blood PD samples, contrary to the expression trend already reported in AD. We found this observation fascinating, and we assume ABCA7 has excellent potential to be the key marker in PD.

Meanwhile, we also identified that many transcription factors are involved in PD pathological process. Among them, AP1 and SF1 are in the highest priority position of the enriched representative terms. Activator protein 1 (AP1) has been shown to induce neuroinflammation in PD [35]. When combined with the aberrant expression of inflammation-related genetic variables in this study, AP1 may be involved in regulating these factors. Splicing factor SF1, which is associated with the ATP-dependent formation of the spliceosome complex [36], is essential for early spliceosome assembly but not for all pre-mRNA splicing. It has also been found to operate as a transcriptional suppressor [37]. SF1 controls alternative splice site selection in mammalian cells and can control exon inclusion either positively or negatively [38]. SF1 was first discovered to be a protein necessary for pre-spliceosome formation [39]. Alternative signals may change a protein's structure or function within the cell. Thus, the wide variety of proteins that may precisely tailor cellular function to the condition of the cells' biology is produced through alternative splicing [40]. It has been reported that SF1 is associated with aging. For example, the knockdown of human spliceosomal component SF1 in the C. elegans ortholog was sufficient to abolish lifespan extension caused by dietary restriction in C. elegans, but did not shorten wild-type lifespan. So far, no research has conclusively shown that SF1 has a role in the course of neurodegenerative diseases, including PD. In this study, we propose that SF1 is implicated in PD control and has potential research relevance.

However, the limitation of this research is that we did not conduct long-term research to see if gene expression levels can track clinical development, including the transcription factors and peripheral clinical blood PD markers that we validated in this study. We also should have investigated and confirmed the precise processes of these indicators in the PD regulation process in PD animal and cell models.

## 5. Conclusion

We performed the bioinformatic analysis with multimodal methods and identified 1045 genes expressing differently in PD than the healthy control group in 4 datasets. The differentially expressed genes were then further validated in clinical settings, yielding a total of 8 downregulated genes and 13 upregulated genes. They could be promising markers for discriminating the PD and potential factors that may affect PD development.

## Figures and Tables

**Table 1:** Primer sequences used.

Genes	Primer sequences, 5′–3′ forward	Primer sequences, 5′–3′ reverse
G6PD	TGCCTTCCATCAGTCGGATACAC	TGGTGGGGTAGATCTTCTTCTTGG
GRID2	TGCGGATTCGATCATTCACA	GTGCGAAATACCTCATCATCCTT
MDH2	AGCACCGGAAGAGTCGCT	CTTCCCCAGCTGTTCTCTGAGG
RIPK2	CGCCTCTGGCACTGTGTCGT	CGTGACTGTGAGAGGGACAT
CUL4B	CCTGGAGTTTGTAGGGTTTGAT	GAGACGGTGGTAGAAGATTTGG
BCL6	AATGAGTGTGACTGCCGCTTCT	CACCGGTATGGACGGTCTTG
MRPS31	CTCCACAGAATCCCGGCATTT	ACTGGTCAACTTTCTTGCTACAG
GPI	CCAAGTCCAGGGGCGTG	CTTGTTGACCTCTGGCATCACA
MAP2K1	GCACCCGCTGAAGGCAG	TTCTGCAAGGCCTCCAAGTT
MAPK	GGTCTGTTGGACGTTTTTAC	TAGGTTTTAGGTCCCTGTGA
ELAVL1	AGCTACGAATCTCCGACCAC	CGTTATCCCATGTGTCGAAGAA
RAB14	TATGGCTGATTGTCCTCACACA	CTGTCCTGCCGTATCCCAAAT
KLF9	GGGAAACCTCCGAAAA	CGTTCACCTGTATGCACTGTA
ARF1	AACACCTTCGCTGTCTGGGATG	GGCAAGTGAGCCTTGATGTGTG
ARFGAP1	GCGCATCCTCATTGCAG	CTTCCTGGTTCTTGGGCTG
ATG7	ACCCAGAAGAAGCTGAACGA	CTCATTTGCTGCTTGTTCCA
ABCA7	GTGCTATGTGGACGACGTGTT	TGTCACGGAGTAGATCCAGGC
E2F2	CCTTGGA GGCTACTGACAGC	CCACAGGTAGTCGTCCTGGT
SFT2D2	GGGACTGGACCCGGAAGA	TTGTCCATTGCGGCCCAG
MAPK7	CAAGAACCTGGCCCTGCTTA	TCCAGGACCACGTAGACAGA
UHRF1	ACACTTGGCTAGTCGTTAATGC	TATGGCCGTCCTCCATCTGT
GAPDH	AGAAGGCTGGGGCTCATTTG	AGGGGCCATCCACAGTCTTC

**Table 2:** Datasets information.

Database	No.	Published date	Samples
GEO	GSE6613	2010	Whole blood
GEO	GSE54536	2014	Whole blood
Proteomexchange	PXD000427	2014	Postmortem substantia nigra
Proteomexchange	PXD022092	2021	Postmortem substantia nigra

**Table 3:** Top 20 clusters with their enriched representative terms (one per cluster). “Count” is the number of genes in the user-provided lists with membership in the given ontology term. “%” is the percentage of all of the user-provided genes that are found in the given ontology term (only input genes with at least one ontology term annotation are included in the calculation). “Log10(*P*)” is the *P* value in log base 10. “Log10(*q*)” is the multitest adjusted *P* value in log base 10.

GO	Category	Description	Count	%	Log10(*P*)	Log10(*q*)
GO:0006091	GO biological processes	Generation of precursor metabolites and energy	111	11.92	-62.71	-58.36
hsa01200	KEGG pathway	Carbon metabolism	62	6.66	-62.25	-58.36
GO:0032787	GO biological processes	Monocarboxylic acid metabolic process	90	9.67	-35.65	-32.14
R-HSA-6798695	Reactome gene sets	Neutrophil degranulation	80	8.59	-34.73	-31.27
ko00020	KEGG pathway	Citrate cycle (TCA cycle)	24	2.58	-30.71	-27.43
GO:0019693	GO biological processes	Ribose phosphate metabolic process	67	7.2	-29.43	-26.19
WP3925	WikiPathways	Amino acid metabolism	35	3.76	-28.63	-25.47
ko00640	KEGG pathway	Propanoate metabolism	22	2.36	-25.68	-22.75
GO:0060627	GO biological processes	Regulation of vesicle-mediated transport	72	7.73	-25.56	-22.66
GO:0032940	GO biological processes	Secretion by cell	87	9.34	-24.09	-21.28
R-HSA-8953897	Reactome gene sets	Cellular responses to stimuli	83	8.92	-22.52	-19.77
R-HSA-422475	Reactome gene sets	Axon guidance	69	7.41	-22.39	-19.66
GO:0010035	GO biological processes	Response to inorganic substance	70	7.52	-22.09	-19.43
GO:0044283	GO biological processes	Small-molecule biosynthetic process	68	7.3	-21.58	-18.95
ko00620	KEGG pathway	Pyruvate metabolism	21	2.26	-21.27	-18.65
WP3888	WikiPathways	VEGFA-VEGFR2 signaling pathway	59	6.34	-20.77	-18.17
GO:0005975	GO biological processes	Carbohydrate metabolic process	69	7.41	-20.62	-18.03
GO:0007005	GO biological processes	Mitochondrion organization	63	6.77	-20.25	-17.66
GO:0051640	GO biological processes	Organelle localization	64	6.87	-20.17	-17.59
ko04721	KEGG pathway	Synaptic vesicle cycle	24	2.58	-19.71	-17.16

**Table 4:** Summary of enrichment analysis in transcription factor targets.

GO	Description	Count	%	Log10(P)	Log10(q)
M9431	AP1 Q6	38	4.1	-15	-12
M1630	TGACCTTG SF1 Q6	37	4	-14	-12
M8812	SF1 Q6	37	4	-14	-11
M1608	NFE2 01	35	3.8	-12	-9.5
M6969	BACH1 01	34	3.7	-12	-9.4
M10220	AP1 01	34	3.7	-11	-9.1
M40742	GTF2A2 target genes	49	5.3	-11	-9
M2054	AP1 Q6 01	33	3.5	-11	-8.7
M9769	CTGCAGY unknown	61	6.6	-10	-8.3
M40770	ATXN7L3 target genes	33	3.5	-10	-8.1
M16482	TTCNRGNNNNTTC HSF Q6	24	2.6	-10	-8
M402	TTCYRGAA unknown	36	3.9	-9.8	-7.8
M29968	FOXE1 target genes	57	6.1	-9.8	-7.7
M2489	ERR1 Q2	31	3.3	-9.6	-7.5
M7806	HSF Q6	27	2.9	-9.4	-7.4
M18894	TCANNTGAY SREBP1 01	43	4.6	-9.3	-7.3
M13237	BACH2 01	31	3.3	-9.3	-7.3
M613	TGANNYRGCA TCF11MAFG 01	33	3.5	-9.2	-7.3
M3403	GTGACGY E4F1 Q6	52	5.6	-8.9	-6.9
M8004	TGASTMAGC NFE2 01	25	2.7	-8.7	-6.8

## Data Availability

The datasets generated/analyzed during the current study are available.
